# Rhythmic Calcium Events in the Lamina Propria Network of the Urinary Bladder of Rat Pups

**DOI:** 10.3389/fnsys.2017.00087

**Published:** 2017-12-11

**Authors:** Thomas J. Heppner, Grant W. Hennig, Mark T. Nelson, Margaret A. Vizzard

**Affiliations:** ^1^Department of Pharmacology, The Robert Larner, M.D. College of Medicine, University of Vermont, Burlington, VT, United States; ^2^Department of Neurological Sciences, The Robert Larner, M.D. College of Medicine, University of Vermont, Burlington, VT, United States

**Keywords:** ATP, TRPV4, PDGFRα, CPA, wavefront, network, postnatal development

## Abstract

The lamina propria contains a dense network of cells, including interstitial cells (ICs), that may play a role in bladder function by modulating communication between urothelium, nerve fibers and smooth muscle or acting as pacemakers. Transient receptor potential vanilloid 4 (TRPV4) channels allow cation influx and may be involved in sensing stretch or chemical irritation in urinary bladder. Urothelium was removed from rats (P0-Adult), cut into strips, and loaded with a Ca^2+^ fluorescent dye (Fluo-2 AM leak resistant or Cal 520) for 90 min (35–37°C) to measure Ca^2+^ events. Ca^2+^ events were recorded for a period of 60 seconds (s) in control and after drug treatment. A heterogeneous network of cells was identified at the interface of the urothelium and lamina propria of postnatal rat pups, aged ≤ postnatal (P) day 21, with diverse morphology (round, fusiform, stellate with numerous projections) and expressing platelet-derived growth factor receptor alpha (PDGFRα)- and TRPV4-immunoreactivity (IR). Ca^2+^ transients occurred at a slow frequency with an average interval of 30 ± 8.6 s. Waveform analyses of Ca^2+^ transients in cells in the lamina propria network revealed long duration Ca^2+^ events with slow upstrokes. We observed slow propagating waves of activity in the lamina propria network that displayed varying degrees of coupling. Application of the TRPV4 agonist, GSK1016790 (100 nM), increased the duration of Ca^2+^ events, the number of cells with Ca^2+^ events and the integrated Ca^2+^ activity corresponding to propagation of activity among cells in the lamina propria network. However, GSK2193874 (1 μM), a potent antagonist of TRPV4 channels, was without effect. ATP (1 μM) perfusion increased the number of cells in the lamina propria exhibiting Ca^2+^ events and produced tightly coupled network activity. These findings indicate that ATP and TRPV4 can activate cells in the laminar propria network, leading to the appearance of organized propagating wavefronts.

## Introduction

The micturition reflex undergoes marked changes during prenatal and postnatal development but the mechanisms underlying these changes are largely unknown. As the CNS matures during the postnatal period, reflex voiding is brought under voluntary control involving higher brain centers (Capek and Jelinek, 1956; Sugaya et al., 1997; Sillen, 2001; Ng et al., 2007). Injuries of the adult nervous system including spinal cord injury (SCI) can lead to the reemergence of a loss of voluntary control (de Groat et al., 1998). Micturition in neonates of many species is dependent on activation of a spinal reflex pathway triggered when the mother licks the perineal region of the newborn (Capek and Jelinek, 1956; Sugaya et al., 1997; Sillen, 2001; Ng et al., 2007). This reflex pathway is essential to prevent urinary retention and consists of a somatic afferent limb in the pudendal nerve and a parasympathetic efferent limb in the pelvic nerve (de Groat et al., 1998). Human infants have a similar reflex pathway (Sillen, 2001). As the neonate develops, the perineal-to-bladder reflex weakens and is replaced by an inhibitory perineal-to-bladder reflex and the adult form of voiding (Fowler et al., 2008; de Groat and Yoshimura, 2015; de Groat et al., 2015). As the nervous system continues to mature, the spinal micturition reflex is gradually replaced by a spinobulbospinal reflex pathway activated by mechanosensitive afferent nerve activity to evoke micturition beginning in the rat between postnatal (P)16 and P18 (Fowler et al., 2008; de Groat and Yoshimura, 2015; de Groat et al., 2015).

The urinary bladder has two main functions; it must be able to expand to accommodate urine continuously produced by the kidneys and empty rapidly when needed. Storage and elimination functions involve the reciprocal functions of the bladder, urethra and external urethral sphincter, which are controlled by the coordination of the different tissue layers in the bladder wall and organized by complex neural pathways organized in the CNS and PNS (Andersson, 2002, 2004; Merrill et al., 2016). To sustain continuous storage and elimination phases, the urinary bladder is organized into three well-defined layers: the mucosa, muscularis propria, and the adventitia/serosa. The mucosal layer consists of transitional epithelial cells that line the lumen of the bladder and a lamina propria beneath the basement membrane of the epithelial cells (Andersson, 2002, 2004; Merrill et al., 2016). The transitional epithelial cells, termed the urothelium, function not only as an impermeable, nonadherent barrier, but also as a sensory component that is capable of responding to multiple and diverse mechanical and chemical stimuli. The urothelium can also respond to stimuli and release various factors including ATP, acetylcholine and nitric oxide (Birder and Andersson, 2013; Merrill et al., 2016). The barrier and signaling functions of the urothelium can be compromised during injury or inflammation, allowing toxic substances to reach the subepithelial nerve plexus and muscular layers, contributing to urinary urgency, frequency, and pain during voiding. The lamina propria lies between the urothelium and the detrusor and is composed of loose connective tissue, interstitial cells (ICs), vasculature, lymphatic vessels, nerve fibers and nerve terminals and may serve to integrate epithelial and smooth muscle input to maintain normal bladder function (Andersson, 2002, 2004; Birder and Andersson, 2013). The distribution and proximity of structural components of the urinary bladder suggests that reciprocal communication is possible between urothelial cells, components in the lamina propria and detrusor smooth muscles (Birder and Andersson, 2013).

ICs are found in many different tissues but, their role is poorly understood in the urinary bladder. In the gut, the role of a specialized class of ICs, the interstitial cells of Cajal (ICC) has been extensively studied and found to be key to generating slow waves and coordinating motility as well as regulating neurotransmission (Gfroerer and Rolle, 2013). In urinary bladder, despite numerous studies, the role of ICs is unknown. Antibodies against kit (proto-oncogene receptor tyrosine kinase, c-kit) labels ICC in the gut as well as macrophages (Ward et al., 1999). McCloskey (2010) showed kit-positive cells were located in both the detrusor and lamina propria of the mouse bladder (McCloskey, 2010) that had stellate-shaped morphology with numerous branches and containing gap junctions (Sui et al., 2002; McCloskey, 2010). In adult and neonatal rats as well as humans, lamina propria kit-positive cells are extensively linked by connexin (Cx)43 gap junctions to form a syncytium (Sui et al., 2002; Ikeda et al., 2007).

ICs in the lamina propria are sometimes called myofibroblasts based on their ultrastructural characteristics (Wiseman et al., 2003) and may interact with an extensive network of nerve fibers (subepithelial plexus), including afferents, that course through the lamina propria (Gabella and Davis, 1998). Immunohistochemistry as well as electron microscopy indicates a close association between lamina propria cells and nerve fibers (Wiseman et al., 2003; Davidson and McCloskey, 2005; Andersson and McCloskey, 2014). A subpopulation of kit-positive lamina propria cells that express platelet-derived growth factor receptor-α (PDGFRα) were also found in murine bladder (Koh et al., 2012). PDGFRα cells exhibited stellate or spindle-shaped morphology and formed a dense network in the lamina propria (Koh et al., 2012). Although the lamina propria is made up of a heterogeneous population of cells types certain cells may be important modulators of neural activity and form a communication link between the urothelium and detrusor; the importance of which may depend upon age or presence of pathology.

Several transient receptor potential (TRP) channels have been identified in the urinary bladder (Birder and Andersson, 2013; Merrill et al., 2016). These channels comprise a superfamily of non-specific cation channels that are generally, but variably, permeable to Ca^2+^, Na^+^, and K^+^ ions and may act as sensors of stretch and/or chemical irritation in the lower urinary tract. The TRP vanilloid (V) 4 (TRPV4) channel is expressed in different cells of the urinary bladder and allows Na^+^ and Ca^2+^ influx into the cell. Measurements of ionic currents and Ca^2+^ events induced by agonists (4α-PDD, GSK1016790A) or stretch have demonstrated functional expression of TRPV4 in urothelial cells (Gevaert et al., 2007b; Everaerts et al., 2010a,b,c) and detrusor (Thorneloe et al., 2008). TRPV4-KO mice demonstrate an abnormal voiding pattern and fewer voiding contractions compared to controls (Gevaert et al., 2007b) suggesting that TRPV4 channels contribute to normal bladder function as well as bladder pathology (Merrill and Vizzard, 2014; Merrill et al., 2016).

In this study, we identified a heterogeneous network of cells at the urothelial-lamina propria interface of rat pups with diverse morphology and numerous processes that exhibit PDGFRα- and TRPV4-immunoreactivity. Cells in this lamina propria network express spontaneous Ca^2+^ events mediated through the release of Ca^2+^ from internal stores and also respond to TRPV4 agonists with changes in Ca^2+^ signaling. Application of exogenous ATP evoked Ca^2+^ waves that propagate through the lamina propria cell network demonstrating a functional syncytium that may provide a critical communication link between the urothelium and the detrusor smooth muscle to convey sensory information or to affect detrusor contractility.

## Materials and methods

### Experimental animals

Wistar rats (Charles River Canada, St. Constant, Quebec) of both sexes and various postnatal (P) ages (P0-Adult) were used in these studies. The University of Vermont Institutional Animal Care and Use Committee approved all experimental protocols involving animal use. Animal care was under the supervision of the University of Vermont's Office of Animal Care Management in accordance with the Association for Assessment and Accreditation of Laboratory Animal Care (AAALAC) and National Institutes of Health guidelines. All efforts were made to minimize the potential for animal pain, stress or distress. Tissues were harvested after euthanasia by decapitation or isoflurane overdose followed by thoracotomy.

### Tissue preparation

Urinary bladders were removed from adult rats (*n* = 13) and rat pups (*n* = 40, *P* ≤ 21) of both sexes and placed in cold HEPES solution consisting of (mM): 134 NaCl, 6 KCl, 10 glucose, 10 HEPES, 1 MgCl_2_, 2 CaCl_2_, 10 glucose and adjusted to pH 7.4 with NaOH. The urothelium was removed from the detrusor and carefully cleaned of lamina propria with sharpened forceps until all visible lamina propria was removed. The cleaned urothelial sheet was placed in buffered 4% paraformaldehyde (pH 7.4) for immunohistochemistry or placed in a special chamber (PSS) for Ca^2+^ imaging studies.

### Immunohistochemistry and visualization

Urothelial tissue sheets were blocked normal goat serum in PBS (pH 7.4) containing 20%, 0.2% Triton X-100 for 2 hr at room temperature and the primary antibody was applied in PBS containing 4% normal goat serum, 0.2% Triton X-100 overnight at 4°C. The following primary antibodies were used: rabbit anti-TRPV4 (1:1K; Abcam, catalog #ab39260) (Merrill et al., 2012), rabbit anti-TRPV4-ATTO-550 (1:500; Alomone Labs, Jerusalem, Israel, catalog #ACC-034-AO), rabbit anti-PDGFRα (1:8K; Thermo-Fisher Scientific, Waltham, MA, catalog #701142), rabbit anti-PDGFRα (1:1K; MyBioSource, Inc. San Diego, CA, catalog #MBS821212). The urothelial tissue sheets were washed in PBS (pH 7.4) containing 0.1% BSA, 0.1% Triton-X-100, 4 X for 15 min each at room temperature. Washed tissue sheets were incubated for 2 h at room temperature in species-specific secondary antibodies. The tissue was washed 3 X for 10 min each in PBS and mounted with mounting medium (Polysciences, Warrington, PA). Immunoreactivity that was greater than the background level in experiment-matched negative controls (preabsorbed antigen peptide; see below) was considered positive. Non-specific staining was assessed by preabsorption treatment with 10^−6^ M of the antigen peptide when available [blocking peptide for TRPV4, Abcam, Inc., (catalog #ab39471); blocking peptide for PDGFRα, MyBioSource, Inc., (catalog #MBS822450)]. Use of the TRPV4 or PDGFRα blocking peptide eliminated immunostaining (data not shown) in tissue sheets. Specificity of TRPV4 expression was also confirmed in TRPV4 null mice (Dr. Kevin Thorneloe, GSK, Philadelphia, PA) (data not shown) (Merrill et al., 2012; Girard et al., 2013). Urothelial tissue sheets were examined under an Olympus fluorescence microscope. Cy3 was visualized with a filter with an excitation range of 560–596 nm and an emission range of 610–655 nm. Digital images were obtained using a charge-coupled device camera (MagnaFire SP, Optronics, Optical Analysis, Nashua, NH) and LG-3 frame grabber (Optical Analysis). Additional images were collected with a Nikon C2 confocal system with a Plan Apo 40X DIC H oil objective with 1.0 NA. Samples were scanned at 1024X1024 resolution using the 488 or 561 nm laser lines, with green and red detectors collecting signal at 525/36 or 605/52 nm, respectively.

### Ca^2+^ imaging

To detect Ca^2+^ events, the urothelial-lamina propria sheets were loaded for 90 min (37°C) with a Ca^2+^ sensitive fluorescent dye, either Fluo-2 AM leak resistant (TEFLabs, Austin, Texas) (10 μM) or Cal 520 (AAT Bioquest, Inc., SunnyVale, California) + pluronic acid (2.5 mg/ml) in HEPES solution. All experiments were conducted in physiological saline solution (PSS) (35–37°C) consisting of (mM): 119 NaCl, 4.7 KCl, 24 NaHCO_3_, 1.2 KH_2_PO_4_, 2.5 CaCl_2_, 1.2 MgSO_4_, 7 glucose and constantly bubbled with Biological Gas (5% CO_2_) to maintain pH at 7.4. The urothelium was visualized with a 60X water immersion (NA 1.2) fluorescent objective. Images were collected with a Noran Oz laser scanning confocal microscope or with a Yokogawa CSU-W1 spinning disk confocal microscope housed in the Imaging/Physiology Core (Larner College of Medicine at The University of Vermont), at a rate of 16–30 images/s. Fluo-2 or Cal 520 were excited at 488 nm, and the emitted fluorescence collected at >500 nm. Imaging fields were 133 × 133 μm (512 × 512 pixel). Ca^2+^ events were initially visualized offline using software developed in our laboratory by Dr. Adrian Bonev. For some experiments Tetramethylrhodamine (TRITC 1 mg/ml; ThermoFischer Scientific) was incubated with fluo-2 AM leak resistant Ca^2+^ sensitive dye for 90 min (35–37°C). TRITC was taken up by urothelial cells and provided contrast to Fluo-2 loaded cells. The tissue was visualized using a Yokogawa CSU-W1 spinning disk confocal microscope housed in the Imaging/Physiology Core. TRITC was excited at 561 nm and emitted fluorescence was collected using a 525/50 filter.

### Data analysis

Detailed analysis of Ca^2+^ events in lamina propria cells was made using custom-written software (Volumetry G8e/G9: Grant Hennig). To localize cells that had Ca^2+^ activity, movies were differentiated (Δt = ±1 s), then frame averaging (±0.15 s) and Gaussian smoothing (3 × 3: sd = 1.0) were applied. The processed movies were thresholded at the level at which noise particles (granular frame by frame fluctuations in intensity) occupied 1% of the total spatio-temporal volume (Drumm et al., 2017). This equates to an imaging signal to noise ratio of >14 dB. Particles less than 15 pixels (1 μm^2^ @ 60x: 9 μm^2^ @ 20x) in total area were filtered out and calcium transient particle (PTCL) files were created. To filter spurious noise particles, the spatial overlap between particles on successive frames was tabulated, then an existence filter was applied that preserved particles that had a continuous spatial overlap for more than 0.25 s. Ca^2+^ transient particles that met or exceeded the above conditions were considered to be resolved Ca^2+^ transients. Initiation sites of Ca^2+^ transients were flagged by choosing the first appearance of a resolved Ca^2+^ transient.

#### Cellular activity masks

The refined Ca^2+^ transient particles were summed throughout the movie to create a prevalence map of activity (as % of recording time a resolvable Ca^2+^ event was present). Ca^2+^ transient prevalence maps were thresholded (>0.5% activity during recording) and bitMasks created from which intensity traces were calculated in the original recording.

#### Ca^2+^ transient characteristics

The interval, duration, amplitude, and rise/decay times of lamina propria cells Ca^2+^ transients were calculated from intensity traces calculated within cell bitMasks. To measure the overall effect of drugs on cells in the lamina propria network, we integrated the area of resolvable Ca^2+^ transient PTCLs over the course of the recording period (see **Figures 4–6**).

#### Propagation

The pattern of Ca^2+^ transients in lamina propria cells ranged from apparently random to well demarcated wavefronts. To quantify the degree of coordination of Ca^2+^ activity in lamina propria cells, the temporal order of every resolvable Ca^2+^ transient in cells within the FOV was determined, then for each sequential pair of events, the (i) time delay, (ii) distance of separation and (iii) angle of between sites was calculated. Any bias in the number of “next-to-fire” angles in a particular direction demonstrate a non-random pattern of activation and was used to gauge relationships between Ca^2+^ transients within cells in the lamina propria syncytium. Random patterns of activation have a little bias (equal representation of all angles between cells in the activation sequence), where as a greater degree of bias (maximum number of events at a particular angle range divided by the minimum number of events at a particular angle range: 360° range; 20° bin size) are indicative of a well-formed wave front.

#### Figure preparation

Images were imported into Photoshop 7.0 (Adobe Systems, San Jose, CA) or Powerpoint (Microsoft PowerPoint for Mac 2011, Version 14.7.3, Microsoft Corporation) where groups of mages were assembled and labeled.

### Statistical analyses

Values are expressed as mean ± S.E.M. from *n* rat pups. Statistical comparisons between groups were made using one-way analysis of variance (ANOVA). When F ratios exceeded the critical value (*p* ≤ 0.05), the Dunnett's *post-hoc* test was used to compare groups.

### Drugs

ATP, Cyclopiazonic acid (CPA), GSK1016790 [*N*-((1*S*)-1-3-hydroxypropanoyl)-1-piperazinyl]carbonyl}-3-methylbutyl)-1-benzothiophene-2-carboxamide] and GSK2193874 [3-([1,4′-Bipiperidin]-1′-ylmethyl)-7-bromo-*N*-(1-phenylcyclopropyl)-2-[3-(trifluoromethyl)phenyl]-4-quinolinecarboxamide] were purchased from Sigma (St. Louis, Missouri, USA), dissolved in PSS, and superfused over the urothelial preparation.

## Results

### Morphology and neurochemistry of cells in lamina propria network

**Figure 1:**
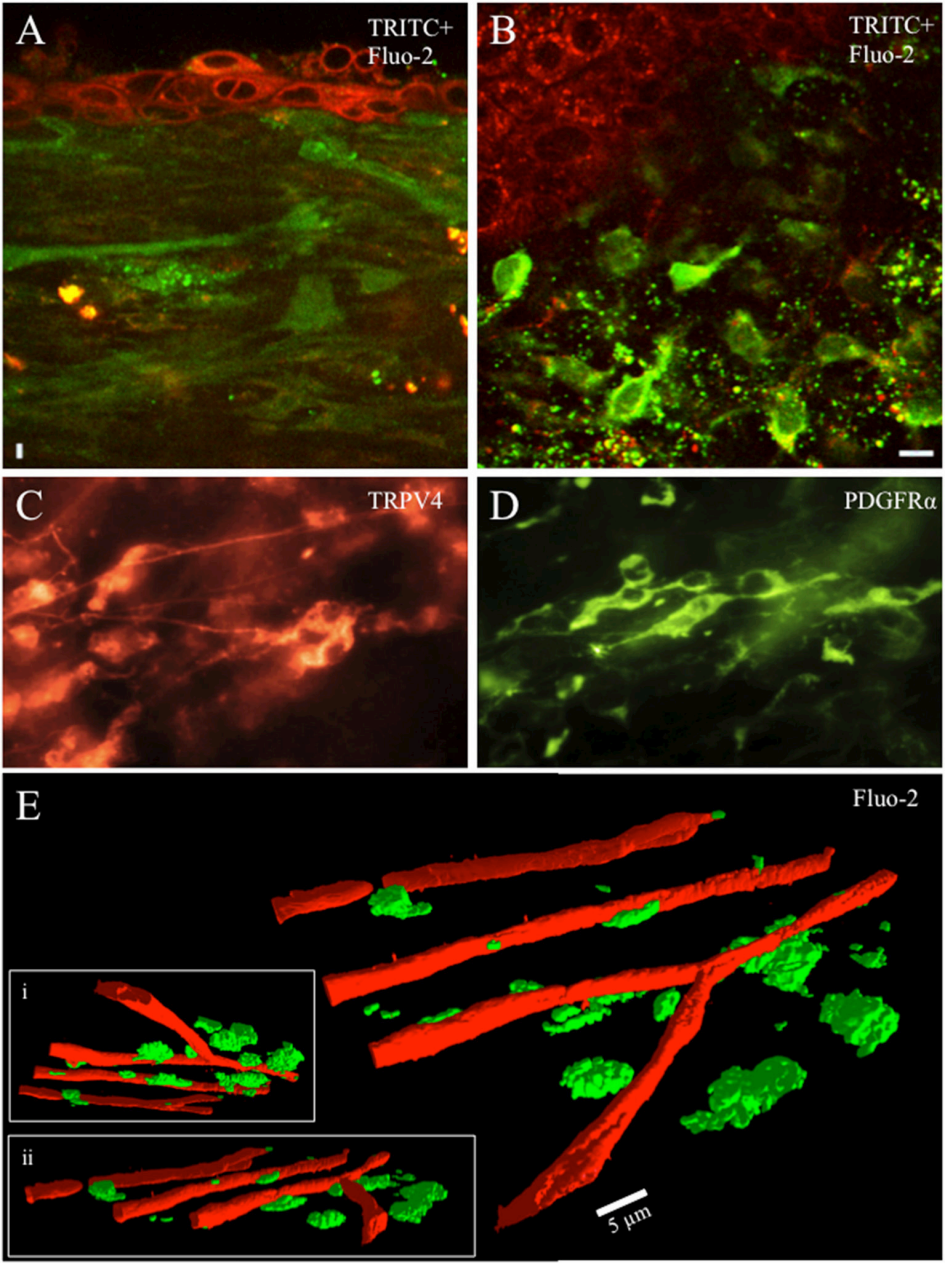
Whole mounts of urinary bladder tissue were prepared at the urothelial-lamina junction and contained a dense network of capillaries, small blood vessels and cells from rat pups aged ≤ P21. Capillaries, blood vessels and cells were loaded with the fluorescent dye (Fluo-2, green) **(A**,**B,E)**. Tetramethylrhodamine (TRITC; red) was picked up by urothelial cells and provided contrast to Fluo-2 loaded cells **(A**,**B)**. The cellular network in the lamina propria was heterogeneous with numerous small round, stellate or spindle shaped cells (10–20 μm) with multiple processes projecting from the soma in rat pups aged *P* ≤ 21. Numerous cells in the lamina propria exhibited PDGFRα-immunoreactivity (IR) whereas fewer cells exhibited TRPV4-IR **(C,D)**. At the urothelial-lamina propria junction, a dense capillary network was in close proximity to the network of cells expressing PDGFRα- and TRPV4-IR **(E)**. Capillaries were isolated using their signature, long tubular shape in the PTCL analysis software and recolored red to distinguish them from lamina propria cells. 3-D images of the capillary network and the lamina propria cell network at different angles of rotation **(E**,**i**,**ii)**. Calibration bar in **(B)** represents 10 μm **(A**,**B)**, 15 μm **(C**,**D)**. Calibration bar in **(E)** represents 5 μm.

In the lamina propria, just below the junction with the urothelium lies a dense network of capillaries, small blood vessels and cells. When incubated with calcium reporter dyes, capillaries, blood vessels and cells readily loaded with the fluorescent dye (Figures 1A,B). Cells in this region were heterogeneous in morphology and most abundant from tissue preparations prepared from rat pups aged ≤ P21. Cells were small in diameter (10–20 μm) and exhibited round, stellate or fusiform morphology with projections, sometimes numerous. In the whole mount preparations cleared of urothelium, numerous cells exhibited PDGFRα-immunoreactivity (IR) whereas fewer cells exhibited TRPV4-IR often occurring in small clusters (Figures 1C,D). The lamina propria cells were in close proximity to the suburothelial nerve plexus, which, in turn, is near the lamina propria capillary network and served as a reliable, readily identifiable landmark allowing us to focus on the same urothelial-lamina junction region between preparations (Figure 1E). Tissue preparations (92.3%) isolated from rat pups aged >P25 exhibited few, if any, cells in the urothelial-lamina propria junction. Because cells in the urothelial-lamina junction that loaded with the calcium reporter dyes were most numerous from rat pups aged ≤ P21, recordings and analyses of spontaneous and evoked calcium events were restricted to this postnatal period.

### Ca^2+^ transients and waveform analyses in the lamina propria network

**Figure 2:**
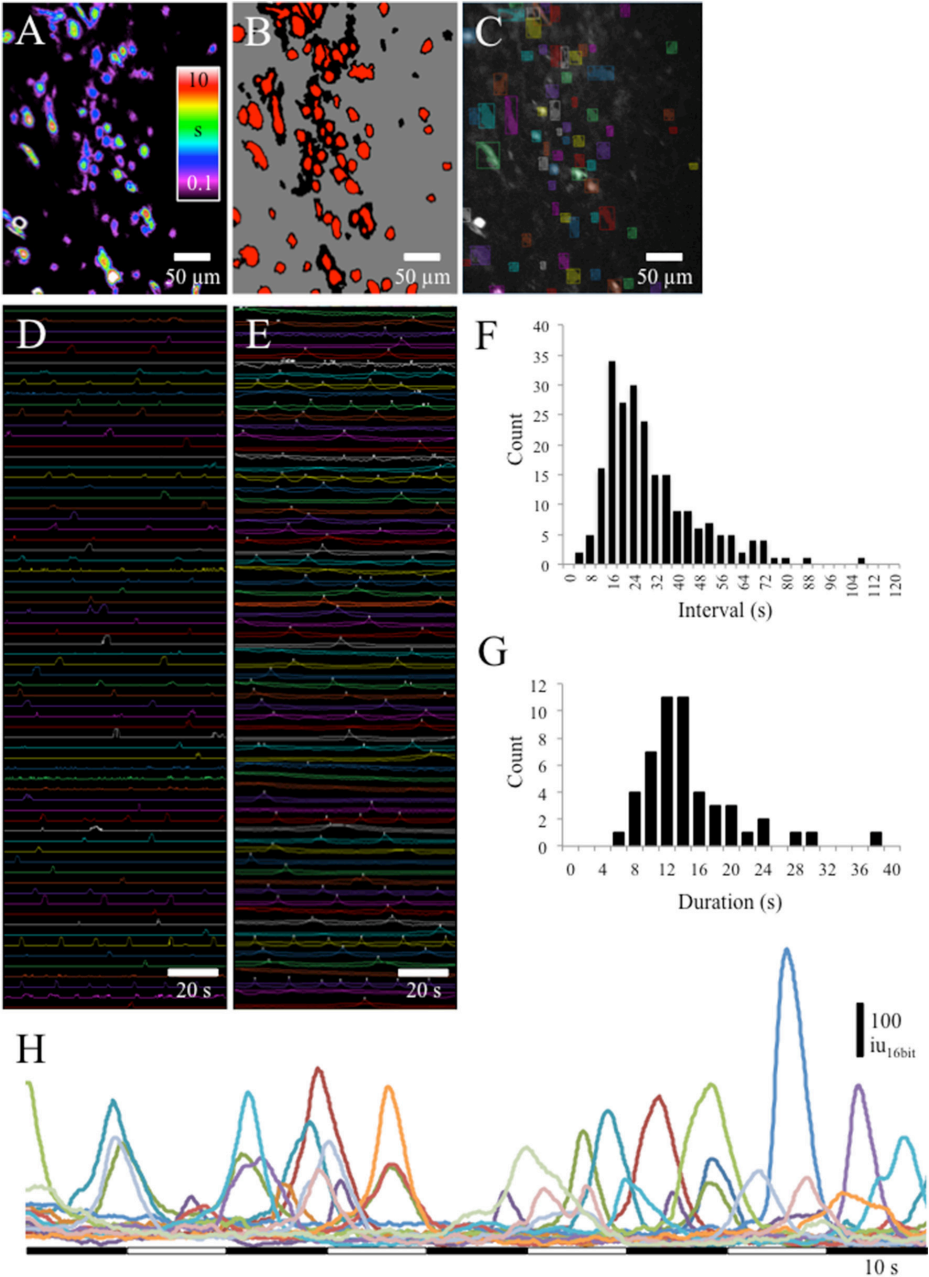
**(A)** Prevalence maps of Ca^2+^ transient PTCLs were thresholded **(B)** to create ROI bitMasks **(C)** to measure PTCL area **(D)** and Ca^2+^ induced fluorescence **(E)** from the original recording. **(F)** Interval histogram shows slow frequency of Ca^2+^ transients in lamina propria cells (average interval = 30 ± 8.6 s; *n* = 4, *o* = 223). **(G)** Duration of Ca^2+^ transients in lamina propria cells was, on average, 15.6 ± 2.4 s (*n* = 56 cells from *n* = 7 different experiments). **(H)** Examples of Ca^2+^ transients in 15 lamina propria cells. Ca^2+^ transients had long durations (5–10 s) and a near symmetrical upstroke and downstroke phases.

At low magnification (20x) Ca^2+^transients could be observed in many cells from lamina propria networks in urothelium sheets from postnatal rats (Figures 2A–E). Ca^2+^ transients occurred at a low frequency with an average interval of 30 ± 8.6 s (Figure 2F; see Supplemental Data, Video). Waveform analyses of Ca^2+^ transients in cells in the lamina propria network revealed long duration Ca^2+^ events (Figure 2G) with slow upstroke and downstroke phases (Figure 2H). Ca^2+^ transient waveform characteristics were calculated from control recordings in 56 cells from 7 different experiments (Table 1).

**Table 1:** Ca^2+^ transient waveform characteristics.

Total Duration (s)	15.55 ± 2.35
AMP (iu)	322.34 ± 79.14
MAXSLP (iu_16_.s^−1^)	197.26 ± 55.40
MINSLP (iu_16_.s^−1^)	−102.93 ± 24.85
MAXLINSLP (iu_16_.s^−1^)	52.75 ± 12.59
MINLINSLP (iu_16_.s^−1^)	−40.95 ± 10.50
RISING TAU (s)	2.36 ± 0.65
FALLING TAU (s)	4.34 ± 1.01
DURATION at 37% of MAX (s)	6.71 ± 1.43
DURATION HALFMAX (s)	5.29 ± 1.27
AUC_ZERO_START (iu.s)	2, 095.60 ± 848.67
AMP (dB)	40.88 ± 1.77
Rising Falling Tau Ratio	0.59 ± 0.11

*Data are presented as Mean ± S.E.M. (n = 56 from 7 experiments)*.

*Amp, Amplitude measured from peak Ca^2+^ transient to start of Ca^2+^ transient; MAXSLP, Maximum Slope (velocity rising phase) measured in iu_16_.s^−1^; MINSLP, Minimum Slope (decay velocity of falling phase) measured in iu_16_.s^−1^; MAXLINSLP, Maximum Linear Slope (measured from start of transient to peak); MINLINSLP, Minimum Linear Slope (measured from peak to end of Ca^2+^ transient); Rising Tau, the time at which Ca^2+^ intensity had increased to 36.8% of its peak value; Falling Tau, the time at which Ca^2+^ intensity had dropped to 63.2% of its peak value; Duration_Eulers, the duration between the rising and falling Tau points; Duration HALFMAX, Duration at half maximum amplitude; AUC_ZERO_START, area under the curve using the start of the transient as the zero point; AMP (dB), This is amplitude of peak Ca^2+^ transient intensity expressed as a signal to noise ratio. The STDEV of the intensity fluctuation before a Ca^2+^ transient was measured, then the ratio of the maximum amplitude to the “noise” (stdev) was calculated using the formula: Amplitude (dB, decibels) = log_10_(maxAmp/STDEV pre-transient) × 20*.

*RisingFallingTauRatio, This parameter was calculated by dividing the rising Tau value by the falling Tau value*.

### Ca^2+^ activity in cells in the lamina propria network

**Figure 3:**
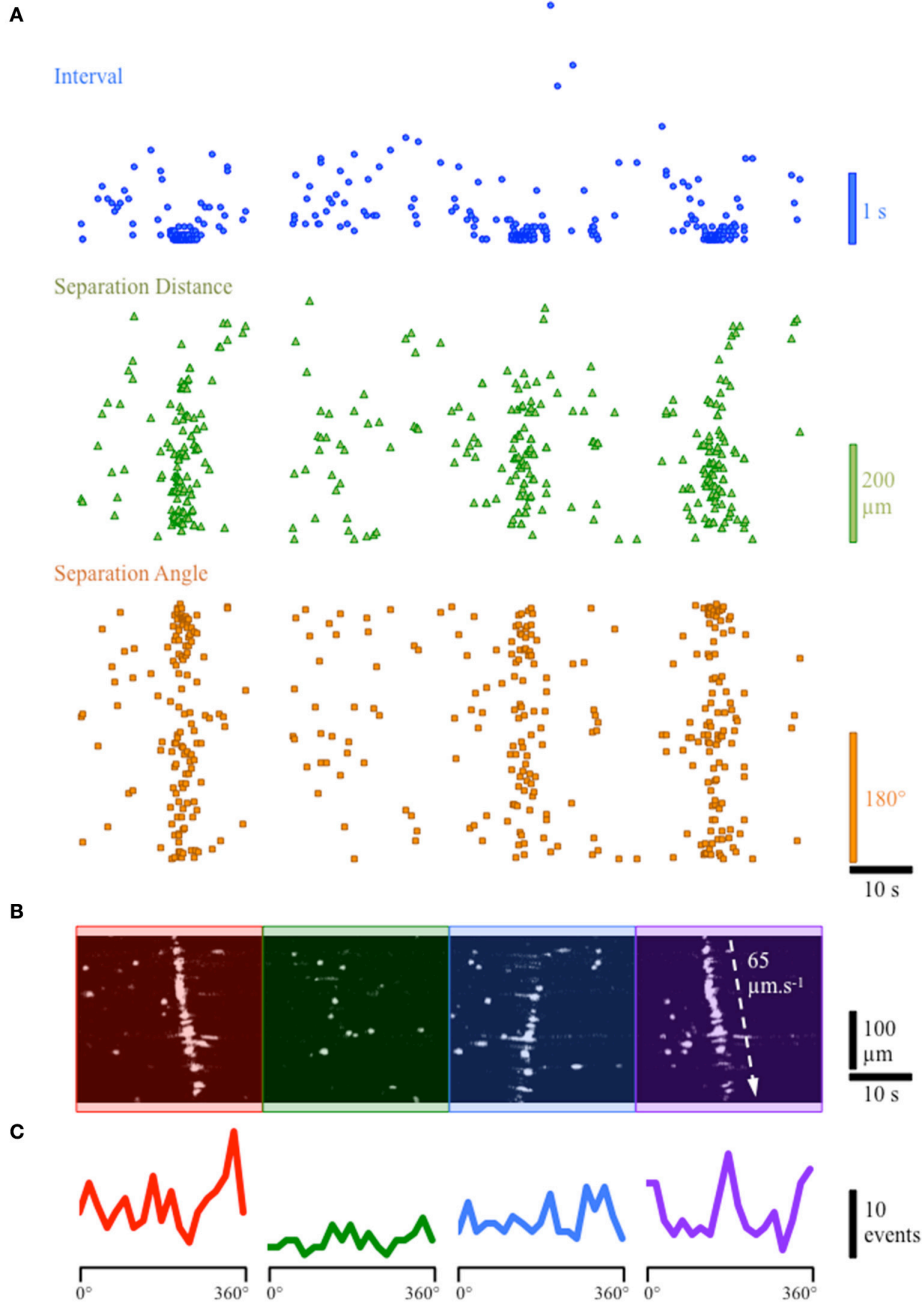
Example of tightly and loosely coupled Ca^2+^ network activity in a continuous recording from the lamina propria cellular syncytium. **(A)** Plot of the firing characteristics of lamina propria cells showing (i) the interval (blue dots), (ii) the distance of separation (green triangles) and (iii) the angle (orange squares) between lamina propria cells in the forward firing sequence. For interval and angle, the lowest values are zero; for separation distance, the lowest value is 1.5 μm. **(B)** Spatio-temporal map of lamina propria cells firing showing 4 network firing events denoted by red, green, blue and purple overlays. **(C)** Histograms of the frequency of angles between next-to-fire cells (forward sequence) during the 4 network firing events. The first and last network firing events show a high degree organization (**B**: red and purple overlays) with a cluster of small delays (**A**: blue dots) between firing of cells and a strong bias for next-to-fire cells to occur at specific angles (**C**: red & purple lines; 160 & 340°) corresponding to the direction of the wavefront (90° to the propagation direction). The second network event (**B**: green overlay) does not show a tightly organized network firing sequence, and has variable delays (**A**: blue dots) with little bias in the angle between next-to-fire cells (**C**: green line). The third event (**B**: blue overlay) in which the wavefront propagates in the opposite direction, shows partially coupled network activity with some bias in the angle between next-to-fire cells but does not involve all of the activateable cells in the field of view (FOV).

During continuous recordings from the lamina propria network, we observed slow propagating waves of activity in which numerous cells in the lamina propria displayed robust, prolonged Ca^2+^ transients (Figures 3A–C). Spatio-temporal maps of the apparent propagation of activity among cells in the lamina propria permitted the determination of any bias in angles between cells in the activation sequence and an estimation of the overall velocity of spread (Figure 3A). In preparations where propagating network Ca^2+^ events were present, the velocity of propagation was consistent at 60–70 μm/s, even though the direction of propagation was often variable. To determine the overall degree of coupling between active lamina propria cells, the bias in angles between lamina propria cells in the activation sequence was measured and averaged 10.65 ± 1.63 (*n* = 8), indicating lamina propria cell activity was not occurring randomly and had a defined direction of propagation regardless of whether the wavefront was loosely or tightly coupled. Spatio-temporal maps demonstrated varying degrees of coupling including tight, partial or loose organization of the lamina propria network are presented in Figures 3B,C. The propagating Ca^2+^ waves illustrate the ability of the lamina propria network to act as a functional syncytium.

### TRPV4 activation of lamina propria network

**Figure 4:**
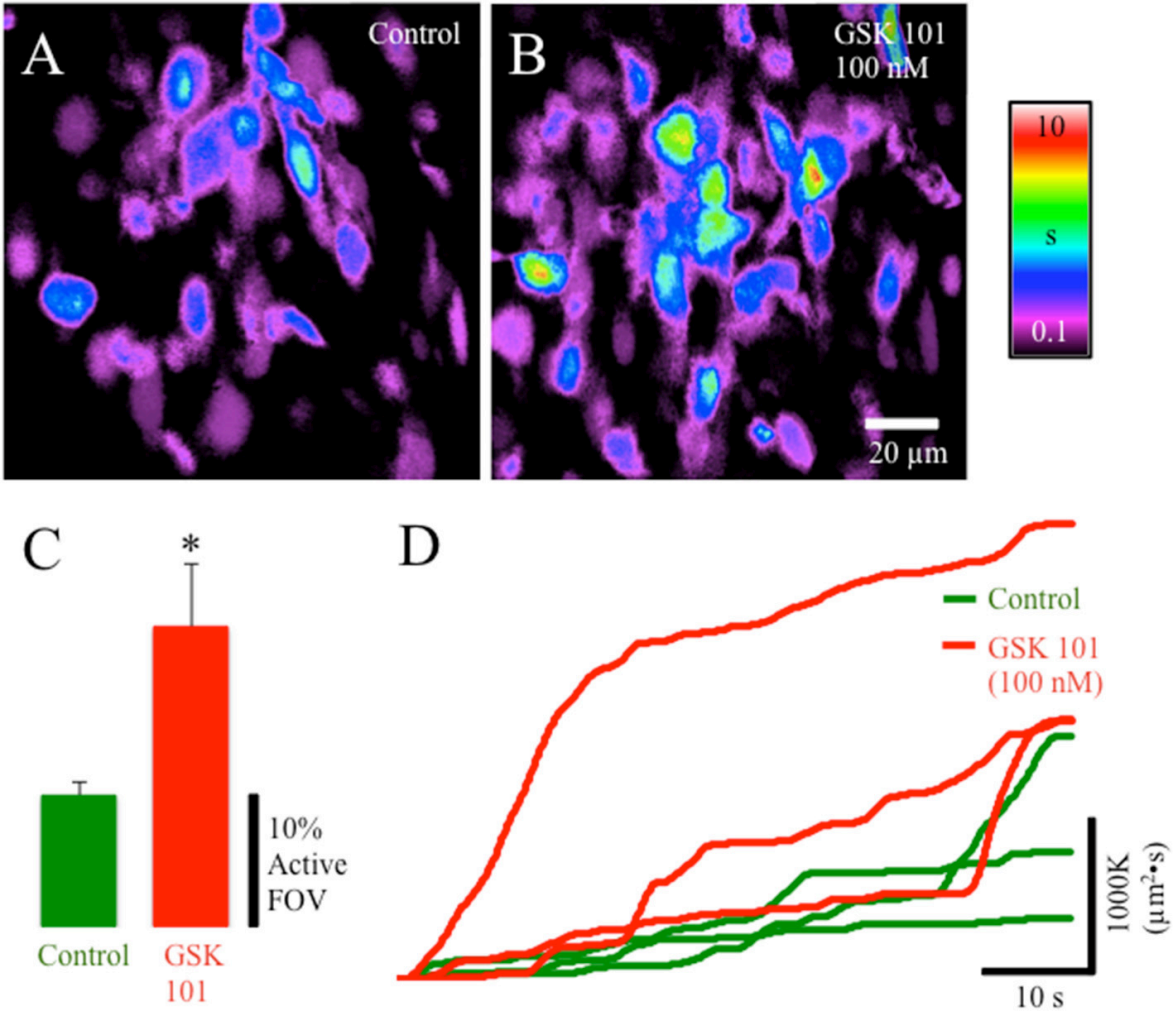
Composite prevalence maps of **(A)** lamina propria cell control activity (*n* = 3 @ 60x) and **(B)** lamina propria cell activity after the addition of GSK1016790 (100 nM). The addition of GSK1016790 increased the time lamina propria cells were active **(B)** and significantly increased the number of cells in the FOV that had active Ca^2+^ events (**C:** ^*^, *p* ≤ 0.038; *n* = 4). **(D)** Traces from individual experiments showing integrated Ca^2+^ activity in control conditions (green lines) and after the addition of GSK1016790 (100 nM: red lines). Notice the periodic increase in rate of integrated Ca^2+^ activity corresponding to coupled lamina propria network events (e.g., propagating wavefronts—see Figure 3).

**Figure 5:**
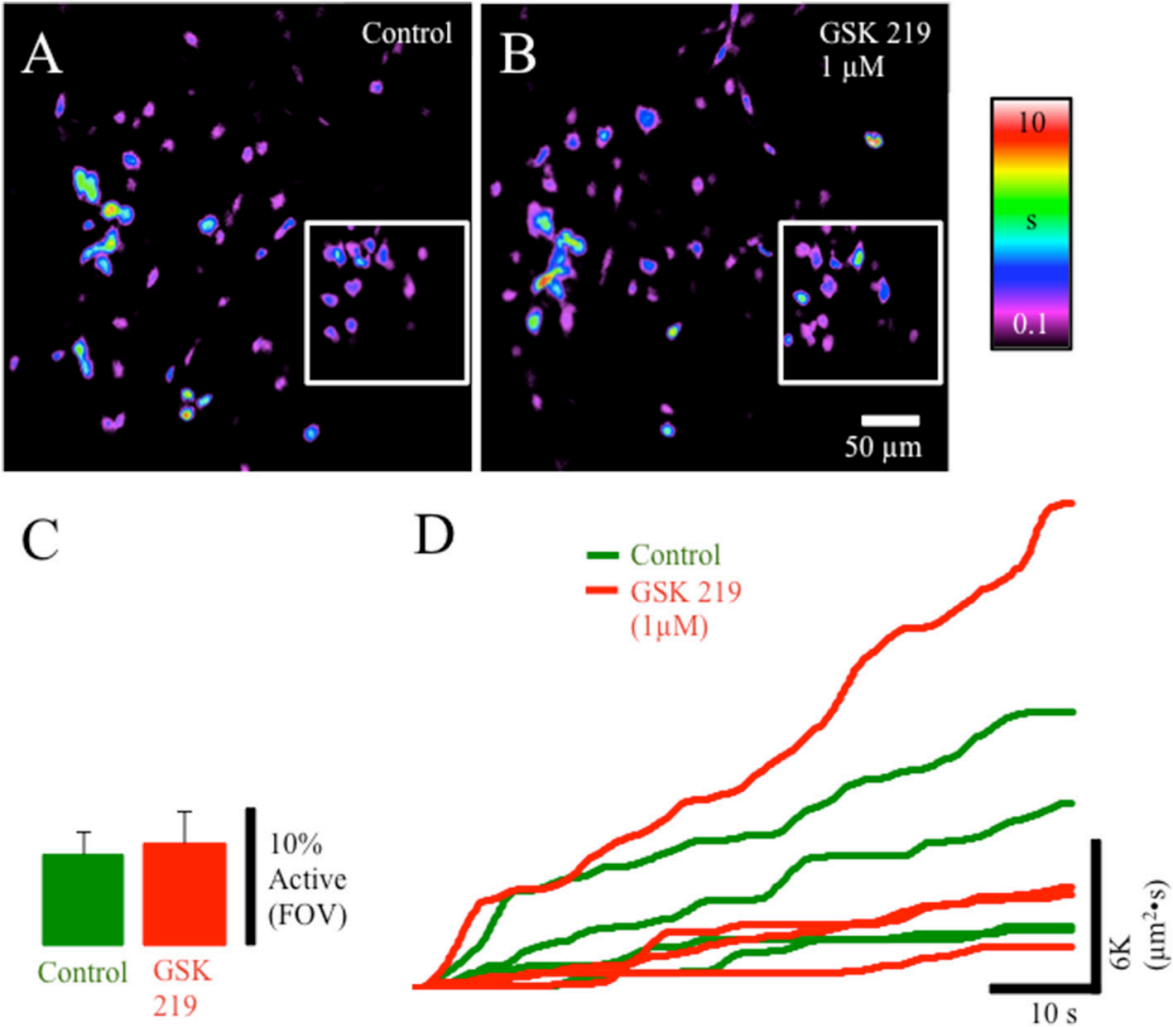
Composite prevalence maps of **(A)** lamina propria cell control activity (*n* = 2 @ 20x; inset *n* = 2 @ 60x) and **(B)** lamina propria cell activity after the addition of GSK2193874 (1 μM). The addition of GSK2193874 did not appreciably alter the number of cells displaying Ca^2+^ transients **(C)**, or the duration **(B)** of Ca^2+^ transients. **(D)** Traces from individual experiments showing integrated Ca^2+^ activity in control conditions (green lines) and after the addition of GSK2193874 (1 μM: red lines).

TRPV4-IR was prevalent in the lamina propria network in postnatal rats. We evaluated the pharmacological activation of TRPV4 with the potent agonist GSK1016790 (Figures 4A–D). GSK1016790 (100 nM) application increased the duration of Ca^2+^ events and the number of cells (*p* ≤ 0.05) with Ca^2+^ events in cells in the lamina propria (Figures 4A–C) observed as an increase in the integrated Ca^2+^ activity (Figure 4D). However, GSK2193874 (1 μM), a potent antagonist of TRPV4 channels, did not affect the number of cells exhibiting Ca^2+^ events or the duration of Ca^2+^ events in cells in the lamina propria (Figures 5A–D) suggesting that TRPV4 channel-mediated Ca^2+^ influx does not contribute to the basal Ca^2+^ signaling.

### CPA blocks Ca^2+^ events in lamina propria network

**Figure 6:**
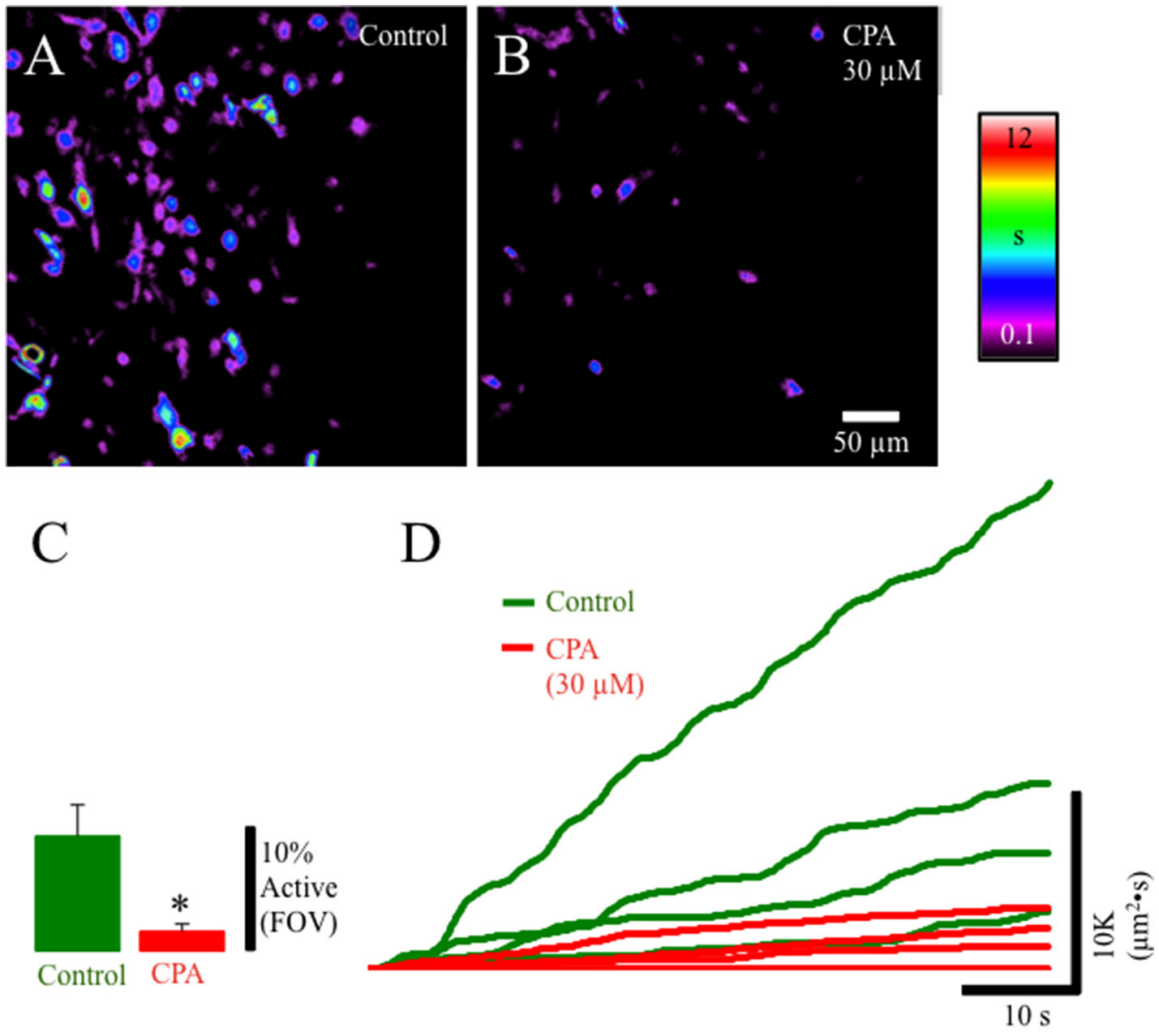
Composite prevalence maps of **(A)** lamina propria cell control activity (*n* = 3 @ 60x) and **(B)** lamina propria cell activity after the addition of CPA 30 μM). The addition of CPA significantly reduced the overall time lamina propria cells were active and significantly reduced the number of cells in the FOV that had active Ca^2+^ events (**C:** ^*^, *p* ≤ 0.01; *n* = 4). **(D)** Traces from individual experiments showing integrated Ca^2+^ activity in control conditions (green lines) and after the addition of CPA (30 μM: red lines).

To identify the potential sources of Ca^2+^ underlying Ca^2+^ transients we used the sarcoendoplasmic reticulum Ca(^2+^) ATPase (SERCA) inhibitor cyclopiazonic acid (CPA; 30 μM). CPA significantly (*p* ≤ 0.01) reduced the overall duration of cell activation and the number of cells exhibiting Ca^2+^ events in the lamina propria network suggesting that Ca^2+^ transients are dependent on release of Ca^2+^ from internal stores (Figures 6A–D).

### ATP activation of Ca^2+^ events in lamina propria network

**Figure 7:**
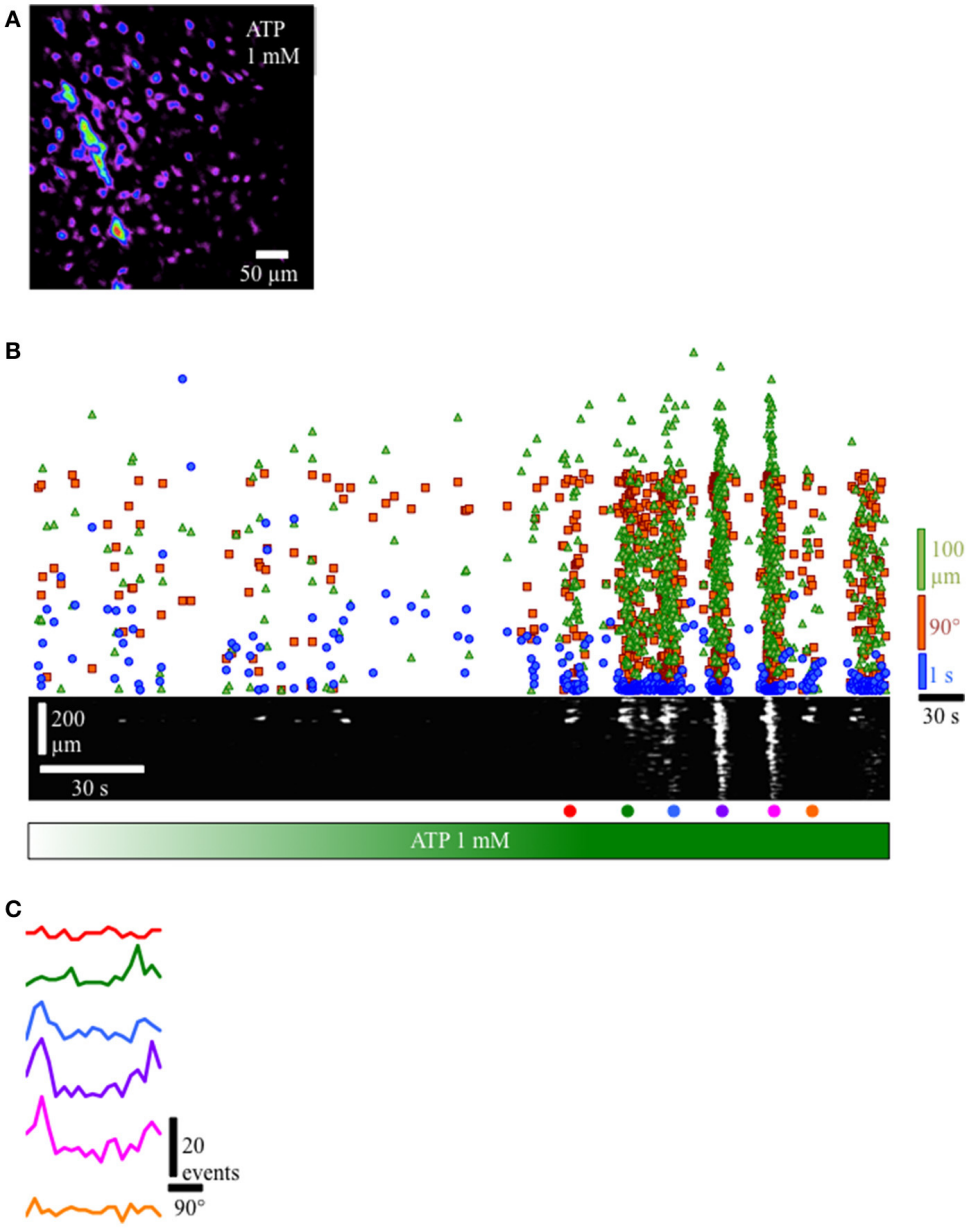
Response of lamina propria cell network to the addition of ATP (1 mM). **(A)** Prevalence map of lamina propria syncytium during the recording (4 min). **(B)** Gradual perfusion of ATP 1 mM (green bar) resulted in increasing number of cells firing, eventually leading to the development of propagating wavefronts as evidenced by the clustering of intervals (blue dots), separation distances (green triangles) and separation angles (orange squares) between next-to-fire cells and coherent, propagating wavefronts in the ST Map (lower panel). **(C)** Angle histograms showing the progression from unorganized activity to well-defined wavefronts based on angle bias. In preparations where propagating network Ca^2+^ events were present, the velocity of propagation was consistent at 60–70 μm/s, even though the direction of propagation was often variable. To determine the overall degree of coupling between active lamina propria cells, the bias in angle averaged 10.65 ± 1.63 (*n* = 8), indicating lamina propria cell activity was not occurring randomly and had a defined wavefront angle and direction of propagation.

Since the lamina propria cells in this study are located immediately beneath the urothelium and are likely exposed to factors produced and released by the urothelium such as ATP, we applied ATP to the lamina propria network. Gradual perfusion of ATP (1 mM) increased the number of cells in the lamina propria exhibiting Ca^2+^ events (Figures 7A–C) that eventually assembled into a propagating wavefront (Figure 7B). This finding indicates that ATP facilitates a behavior transition from apparently random spontaneous Ca^2+^ events in lamina propria cells into well-defined propagating wavefronts.

## Discussion

The current studies confirm the presence of a heterogeneous cellular network in the lamina propria that exhibits spontaneous Ca^2+^ transients that can be loosely or tightly coupled (wavefronts) between cells. In addition, these studies demonstrate several novel findings, including: (i) the predominance PDGFRα- and TRPV4-immunoreactivity in the lamina propria layer from early postnatal rat pups (*P* ≤ 21), (ii) ATP and a TRPV4 agonist activated and increased the number of lamina propria cells that exhibited active Ca^2+^ events, (iii) lamina propria cell activity was not random, with spatio-temporal maps and PTCL analysis demonstrating varying degrees of coupling (e.g., tight, partial or loose organization) and (iv) the coupling of Ca^2+^ activity of cells in laminar propria network could be modified to generate organized, propagating bands of activity (wave fronts) with ATP. These findings are consistent with the hypothesis that lamina propria Ca^2+^ signaling facilitates communication through this syncytial network to other cell types or tissue layers of the urinary bladder; our study shows the spatio-temporal patterning of this potential communication that may affect sensory processing and/or detrusor contractility.

The spinobulbospinal micturition reflex is triggered by tension receptor afferents in the bladder and begins to elicit voiding in the rat between P16-18 (Capek and Jelinek, 1956; Girard et al., 2016) and continues to mature during the postnatal weeks 3–6 (Capek and Jelinek, 1956; Ng et al., 2007). As the CNS matures during the postnatal period in humans, reflex voiding becomes voluntary (2–5 years) and originates in the higher brain centers (de Groat et al., 1998; de Groat and Araki, 1999; Zvarova and Zvara, 2012). Studies in rats (Sugaya et al., 1997) and human infants (Sillen, 2001) demonstrate that supraspinal circuits involved in mature micturition reflex patterns exist in neonatal animals and infants but may not be functional or may function in an inhibitory manner prior to the emergence of a functional, adult micturition pattern (de Groat and Araki, 1999). The micturition reflex continues to mature postnatally to achieve the adult form of reflex voiding. The perineal-to-bladder reflex becomes progressively weaker during the postnatal period and replaced by an inhibitory perineal-to-bladder reflex and the adult micturition reflex. It is hypothesized that upregulation of bulbospinal projections as well as synaptic remodeling in the sacral parasympathetic nucleus in the lumbosacral spinal cord and in the brain contribute to the down-regulation of the perineal-to-bladder reflex. This may be due to competition between supraspinal and segmental neural inputs (Araki, 1994; Araki and de Groat, 1997; de Groat and Araki, 1999).

To function efficiently, the urinary bladder must relax during filling and contract forcefully during micturition. Therefore, communication between the tissue layers in the bladder wall is critical for normal bladder function. Located between the urothelium and detrusor, the lamina propria is frequently described as a functional syncytium and is ideally located to facilitate communication between these tissue layers. Ca^2+^ signals have previously been identified using Ca^2+^ sensitive dyes in isolated lamina propria cells (Wu et al., 2004) and with *in situ* preparations (Gray et al., 2013). Ca^2+^ transients can also be evoked by electrical stimulation (Gray et al., 2013) or with purinergic agonists (Wu et al., 2004). In our study using tissue wholemounts, Ca^2+^ events in the rat pup lamina propria cells had long durations with slow upstroke and downstroke phases, and had a frequency similar to those previously described in the guinea pig (Gray et al., 2013). These Ca^2+^ events were largely dependent on Ca^2+^ release from internal stores because CPA significantly reduced the overall duration of cell activation and the number of cells exhibiting Ca^2+^ events in the lamina propria network. Propagation of Ca^2+^ transients through the lamina propria was first described using a transverse bladder preparation and a photodiode array with a circumscribed sensing area (0.95 × 0.95 mm) for each photodiode. With this system, mechanical or carbachol induced Ca^2+^ transients were found to originate near the urothelial-suburothelial interface with spread to the detrusor (Kanai et al., 2007). This relatively low-resolution system provided evidence that Ca^2+^ transients can propagate through the lamina propria. Using higher resolution, Ca^2+^ spreading between two lamina propria cells via a connecting process was detailed in guinea pig (Gray et al., 2013). In lamina propria cells from rat pup tissue wholemounts, we found Ca^2+^ events often spreading along processes between cells. The ability of the lamina propria network to have a range of Ca^2+^ behaviors ranging from ongoing, loosely-coupled individual cellular activity to where cells were activated nearly synchronously in a particular direction to form a Ca^2+^ wavefront that propagated substantial distances throughout the lamina propria is indicative of flexible coupling between cells in this region (see Figures 3, 7). Although the mechanism underlying the formation of a Ca^2+^ wavefront is unclear, rat pup lamina propria have a high concentration of Cx 43 consistent with the presence of gap junctions (Ikeda et al., 2007). Intercellular Ca^2+^ waves have been observed to propagate at speeds of ~10–20 μm/s, somewhat slower than the 60–70 μm/s we observed in rat pup lamina propria (Leybaert and Sanderson, 2012).

There is increasing evidence that stretch or chemical stimuli of the urothelium initiates the production and release of factors and transmitters including ATP, ACH, and nitric oxide (Birder et al., 1998; Yoshida et al., 2006). ATP (that is not degraded) can stimulate autocrine or paracrine pathways that may convey sensory information to the CNS (Schwiebert and Zsembery, 2003). The transduction pathways within the urinary bladder are affected by receptor subtype expression and their proximity to the urothelium. The tissues and cell types that may contribute to purinergic signaling include nerves in the suburothelial nerve plexus, smooth muscle cells, lamina propria cells including ICs as well as inflammatory cells (Birder, 2005; Birder and Andersson, 2013; Li et al., 2013). For example, ATP was found to activate inward currents (Wu et al., 2004) and Ca^2+^ transients in freshly isolated (Sui et al., 2004) or cultured suburothelial myofibroblasts from human bladder (Cheng et al., 2011). Kit-positive ICs express P2X and P2Y (i.e., P2X3 P2Y2, P2Y4, and P2Y6) receptors and are proposed to form a functional syncytium with smooth muscle cells (Sui et al., 2006; Drumm et al., 2014). In response to ATP, ICCs in the gastrointestinal tract generate P2Y-dependent intracellular Ca^2+^ transients that may then propagate to smooth muscle cells via gap junctions to alter contractility (Drumm et al., 2014). Although the mechanism coupling ICs to sensory activity is unknown, the location of ICs and the responsiveness to ATP suggest they may have a regulatory role in the afferent limb of the micturition reflex (Wu et al., 2004). In the present study, exogenous ATP increased Ca^2+^ transient activity in numerous lamina propria cells that culminated in multiple Ca^2+^ waves propagating through the lamina propria. In addition, these studies demonstrate that exogenous ATP can convert a loosely connected behavior of lamina propria cells to a highly organized behavior with the formation of a Ca^2+^ wavefront. This finding is consistent with the hypothesis that ATP released from urothelium or other tissues (i.e., suburothelial nerves) increases lamina propria Ca^2+^ signaling and facilitates communication through this syncytial network to other cell types or tissue layers (Andersson and McCloskey, 2014). Having a large area of the lamina propria network undergoing near-synchronous activity may provide a much more potent signaling/stimulus to other cell types compared to loosely-coupled patterns of activation of these cells. In addition to the lamina propria network acting as an amplifier of signals/stimuli (Andersson and McCloskey, 2014), we suggest that the lamina propria network is a primary source or originator of coordinated activation of the urinary bladder where it senses and responds to stimuli and communicates this information to the whole urinary bladder. Such a signaling network would be important when neural circuits are not fully mature (i.e., postnatal development) or when compromised by neural injury or disease.

Multiple TRP channels from different subfamilies are expressed in the urinary bladder and have specific tissue distributions in the LUT. These channels are activated by numerous exogenous and endogenous mediators (Skryma et al., 2011; Deruyver et al., 2015) and may have functional roles in the micturition reflex (Andersson et al., 2010; Merrill et al., 2016). Many of these channels are also implicated in bladder disorders including overactive bladder (OAB) and bladder pain syndrome/interstitial cystitis (BPS/IC) (Gevaert et al., 2007a,b; Nilius et al., 2007). Recent studies suggest the involvement of TRPV4 in both normal urinary bladder function and dysfunction (Andersson et al., 2010). TRPV4 was first demonstrated in basal and intermediate urothelial cells (Gevaert et al., 2007a,b) and confirmed by many other studies (Merrill et al., 2016). The functional expression of TRPV4 in urothelial cells has been established following measurements of ionic currents and Ca^2+^ events induced by agonists (4 alpha-phorbol 12,13-didecanoate (4α-PDD), GSK1016790A) or stretch (Kullmann et al., 2009; Mochizuki et al., 2009; Xu et al., 2009). TRPV4 is also expressed in the detrusor smooth muscle; however, transcript levels were found to be approximately 20- to 36-fold higher in the urothelium compared to detrusor smooth muscle (Thorneloe et al., 2008; Xu et al., 2009; Merrill et al., 2012). TRPV4 expression has also been examined in DRG neurons innervating viscera (Yamada et al., 2009; Merrill et al., 2012; Alexander et al., 2013; Girard et al., 2013) but functional evidence is lacking (Alexander et al., 2013). In contrast, the present studies demonstrate functional expression of TRPV4 in lamina propria cells in postnatal rat pups. In wholemount tissue preparations isolated from rat pups aged ≤ P21, TRPV4-IR was observed in cells in the lamina propria that exhibited similar morphology to those expressing PDGFRα-IR. Application of the TRPV4 agonist, GSK1016790, increased the time lamina propria cells were active and increased the number of cells that exhibited active Ca^2+^ events as evidenced by the rate of integrated Ca^2+^ activity. The failure of the TRPV4 antagonist, GSK2193874, to alter the number of lamina propria cells displaying Ca^2+^ transients or the duration of Ca^2+^ transients may indicate that Ca^2+^ influx through TRPV4 channels does not contribute to basal Ca^2+^ signaling in the lamina propria.

The genetic or pharmacological manipulation of TRPV4 has helped to elucidate its physiological role in the micturition reflex. TRPV4 knockout mice exhibited abnormal urine voiding patterns characterized by decreased frequency of voiding contractions and increased frequency of nonvoiding contractions, longer intermicturition intervals and increased total urine volume per void (Gevaert et al., 2007b; Everaerts et al., 2010c). Administration of the TRPV4 agonist, 4α-PDD, to conscious rats resulted in an increase in the amplitude of reflex bladder contractions during cystometry (Birder, 2007). GSK1016790A, a highly selective TRPV4 agonist that is ~300-fold more potent than 4α-PDD, similarly induced bladder hyperactivity *in vivo* in mice (Thorneloe et al., 2008) and rats (Aizawa et al., 2012). Systemic administration of the selective and potent TRPV4 antagonist, HC-067047, decreased voiding frequency and increased bladder capacity in mice and rats following CYP-induced cystitis (Everaerts et al., 2010c) or repeated variate stress (Merrill and Vizzard, 2014). We suggest that the bladder sensory roles of TRPV4 in the normal micturition reflex or following injury or pathology may also be related to TRPV4 expression and function in lamina propria cells.

At the urothelial-lamina propria junction, the network of cells expressing PDGFRα- or TRPV4-IR was sparse and calcium events were not observed. In wholemount preparations from rat pups aged ≤ P21, we consistently identified a dense network of PDGFRα- or TRPV-IR cells in the lamina propria. The reasons underlying these observations are unknown but may reflect the continuing maturation of the micturition reflexes during the early postnatal period. It has previously been speculated that the functional syncytium between ICs and the detrusor smooth muscle is important for coordination of bladder emptying in the early postnatal period because of the absence of mature and functional neural input needed to coordinate the activities of the CNS and PNS including the urinary bladder (Kanai et al., 2007). The current studies in rat pups aged ≤ P21 are consistent with this suggestion as demonstrated by: (i) the predominance of the lamina propria cellular network in early postnatal rat pups; (ii) the ability of ATP and a TRPV4 agonist to activate and increase the number of lamina propria cells that exhibited active Ca^2+^ events; and (iii) the ability of ATP and TRPV4 agonist to increase the rate of integrated Ca^2+^ activity corresponding to coupled lamina propria network events and the formation of propagating wavefronts. Thus, the lamina propria network may have an active role in sensing (e.g., distension) and signaling, perhaps reciprocally, between bladder layers and cell types to achieve coordinated bladder function. The importance of the lamina propria network may depend upon the integrity and maturity of neural pathways that coordinate micturition reflex events.

Altered ATP purinergic signaling in the LUT that include changes in ATP release, expression or density of purinergic receptors and/or expression of ATPases and other ecto-nucleotidases, may contribute to voiding dysfunction, mechanical hypersensitivity and pain (Sun et al., 2001a,b). Increased levels of urinary ATP have been demonstrated in patients with IC (Sun et al., 2001a,b) and overactive bladder (Silva-Ramos et al., 2013; Burnstock, 2014). Primary bladder urothelial cells from these patients also exhibit increased ATP release in response to a variety of stimuli (Sun et al., 2001a; Sun and Chai, 2006). Changes in P2X receptor subtype expression in various bladder tissues have also been demonstrated in patients with IC (Sun and Chai, 2004), detrusor instability (O'Reilly et al., 2002) or bladder outlet obstruction (O'Reilly et al., 2001). In addition, altered protein and transcript expression of TRPV4 in the urinary bladder has been demonstrated during postnatal development and in mice with chronic, urothelial overexpression of NGF (Merrill et al., 2012; Girard et al., 2013). TRPV4 antagonists decrease bladder activity making it a promising target for overactive bladder and other bladder disorders (Birder, 2007; Gevaert et al., 2007b; Merrill et al., 2016). In future studies, it would be of interest to examine the lamina propria network in wholemount preparations from preclinical animal models of bladder dysfunction or SCI (i.e., upper motoneuron injury) to determine if the presence of the lamina propria network at the urothelial-lamina propria junction is changed and/or if spontaneous and evoked Ca^2+^ transients and network activity is altered. A change in the number of cells exhibiting Ca^2+^ events, their pattern of activation, the duration of Ca^2+^ events and/or the ability of mediators (e.g., ATP, TRPV4 agonists) to increase the rate of integrated Ca^2+^ activity corresponding to coupled lamina propria network events could contribute to altered sensory processing (e.g., mechanosensation, pain) and altered urinary bladder function (i.e., hyperactivity). There is precedent for plasticity in the expression of lamina propria cellular network. ICs in the bladder wall following SCI (5 weeks post injury) are decreased, target organ innervation is reduced and the smooth muscle is hypertrophied (Johnston et al., 2012). However, there are differing reports concerning changes in ICs from individuals with bladder dysfunction (e.g., neurogenic and idiopathic detrusor overactivity; McCloskey, 2010). Thus, it would be of interest and potential importance to continue to examine the lamina propria network at the urothelial-lamina propria junction following neural injury, disease or urinary bladder dysfunction to fully understand its functional significance.

## Ethics statement

The studies described from the Vizzard laboratory were performed in accordance with institutional and national guidelines and regulations. The University of Vermont Institutional Animal Care and Use Committee approved all experimental protocols involving animal use. Animal care was under the supervision of the University of Vermont's Office of Animal Care Management in accordance with the Association for Assessment and Accreditation of Laboratory Animal Care (AAALAC) and National Institutes of Health guidelines. All efforts were made to minimize the potential for animal pain, stress or distress.

## Author contributions

Analyzed the data, performed experiments, conceived, discussed and outlined the experimental design: TH, GH, and MV. Wrote the paper, drafted and revised paper: TH, GH, MN, and MV.

### Conflict of interest statement

The authors declare that the research described from the Vizzard laboratory were conducted in the absence of any commercial or financial relationships that could be construed as a potential conflict of interest. The funding entity, NIH, had no role in the studies described including: design, data collection and analysis of studies performed in the Vizzard laboratory, decision to publish or preparation of the review. The contents are solely the responsibility of the authors and do not necessarily represent the official views of NIH.

## Abbreviations

4α–PDD: 4 alpha-phorbol 12,13-didecanoate
Amp: amplitude
ANOVA: analysis of variance
ATP: adenosine 5′-triphosphate
AUC_ZERO_START: area under the curve using the start of the transient as the zero point
BPS: bladder pain syndrome
CNS: central nervous system
CPA: cyclopiazonic acid
Cx43: connexin 43
DRG: dorsal root ganglia
Duration HALFMAX: duration at half maximum amplitude
FOV: field of view
IC: interstitial cystitis
ICs: interstitial cells
ICC: interstitial cells of Cajal
IR: immunoreactivity
KO: knockout
LUT: lower urinary tract
MAXLINSLP: maximum linear slope
MAXSLP: maximum slope
MINLINSLP: minimum linear slope
MINSLP: minimum slope
P: postnatal
P2X: purinergic 2 receptor subtype X
P2Y: purinergic 2 receptor subtype Y
PAR_2_: protease activated receptor 2
PBS: phosphate-buffered saline
PDGFRα: platelet-derived growth factor receptor alpha
PNS: peripheral nervous system
PSS: physiological saline solution
PTCL: calcium transient particle
ROI: region of interest
s: seconds
SCI: spinal cord injury
SEM: standard error of mean
TRITC: tetramethylrhodamine
TRP: transient receptor potential
TRPV4: transient receptor potential cation channel subfamily vanilloid member 4
μm: micrometer.
