# Supplementary material for: Rhythmic Calcium Events in the Lamina Propria Network of the Urinary Bladder of Rat Pups
Source: Front Syst Neurosci. 2017 Dec 11;11:87. doi: 10.3389/fnsys.2017.00087 (PMC5732214; doi:10.3389/fnsys.2017.00087)
Supplement: Supplementary file 3 [file DataSheet1.docx]

**Supplemental Methods**

*Movie Processing*:

To generate Ca2+ transient PTCLs stacks were differentiated (±1s), then frame averaged (±0.15s), then Gaussian Filtered (sd 1.0, 3x3 pixels). Threshold applied such that shot noise size < 15 pixels. Removed particles < 15 pixels in size (mostly noise) then saved as PTCL file.

*Calcium transient particle (PTCL) Processing:*

Size filtered (>25 pixels @20x; >75 pixels @ 60x), PTCL frame overlap calculated then existence filter applied (contiguous PTCL frame overlaps for >= 0.3s). Prevalence maps created by summing flagged particles (units: seconds of prevalence).

*Data Extraction*:

Region of interest (ROI) bitMasks created from Prevalence maps (prevalence > 0.5s: size >25 pixels @20x; >75 pixels @ 60x), then overlaid on original recording to extract time courses of Ca^2+^-induced fluorescence.

*Waveform Analysis*:

Trace smoothing (±0.5s) followed by moving baseline (avg ± 12s) with offset (4%) and peak detection (Max above baseline).

*Parameter Calculation*:

The following Ca^2+^ transient waveform characteristics **(**mean ± S.E.M.; n=56 from 7 experiments) were determined (Table 1): Amp = Amplitude measured from peak Ca^2+^ transient to start of Ca^2+^ transient**;** MAXSLP = Maximum Slope (velocity rising phase) measured in iu_16_.s^-1^**;** MINSLP = Minimum Slope (decay velocity of falling phase) measured in iu_16_.s^-1^**;** MAXLINSLP = Maximum Linear Slope (measured from start of transient to peak)**;** MINLINSLP = Minimum Linear Slope (measured from peak to end of Ca^2+^ transient)**;** Rising Tau = the time at which Ca^2+^ intensity had increased to 36.8% of its peak value**;** Falling Tau = the time at which Ca^2+^ intensity had dropped to 63.2% of its peak value**;** Duration_Eulers = the duration between the rising and falling Tau points**;** Duration HALFMAX = Duration at half maximum amplitude.**;** AUC_ZERO_START = area under the curve using the start of the transient as the zero point**;** AMP (dB): This is amplitude of peak Ca^2+^ transient intensity expressed as a signal to noise ratio.  The STDEV of the intensity fluctuation before a Ca^2+^ transient was measured, then the ratio of the maximum amplitude to the “noise” (stdev) was calculated using the formula:  Amplitude (dB: decibels) = log_10_(maxAmp/STDEV pre-transient) x 20; RisingFallingTauRatio: This parameter was calculated by dividing the rising Tau value by the falling Tau value.
